# How many species are infected with *Wolbachia*? – a statistical analysis of current data

**DOI:** 10.1111/j.1574-6968.2008.01110.x

**Published:** 2008-02-29

**Authors:** Kirsten Hilgenboecker, Peter Hammerstein, Peter Schlattmann, Arndt Telschow, John H Werren

**Affiliations:** 1 Institute for Theoretical Biology, Humboldt University Berlin Berlin, Germany; 2 Department of Biostatistics and Clinical Epidemiology, Charité-Universitätsmedizin Berlin Berlin, Germany; 3 Center for Ecological Research, Kyoto University Otsu, Shiga, Japan; 4 Department of Biology, University of Rochester Rochester, NY, USA

**Keywords:** *Wolbachia*, beta-binomial model, meta-analysis, infection rates

## Abstract

*Wolbachia* are intracellular bacteria found in many species of arthropods and nematodes. They manipulate the reproduction of their arthropod hosts in various ways, may play a role in host speciation and have potential applications in biological pest control. Estimates suggest that at least 20% of all insect species are infected with *Wolbachia*. These estimates result from several *Wolbachia* screenings in which numerous species were tested for infection; however, tests were mostly performed on only one to two individuals per species. The actual percent of species infected will depend on the distribution of infection frequencies among species. We present a meta-analysis that estimates percentage of infected species based on data on the distribution of infection levels among species. We used a beta-binomial model that describes the distribution of infection frequencies of *Wolbachia*, shedding light on the overall infection rate as well as on the infection frequency within species. Our main findings are that (1) the proportion of *Wolbachia*-infected species is estimated to be 66%, and that (2) within species the infection frequency follows a ‘most-or-few’ infection pattern in a sense that the *Wolbachia* infection frequency within one species is typically either very high (>90%) or very low (<10%).

## Introduction

The infection rate of *Wolbachia* is generally estimated to be at least 20% (Werren *et al*., 1995; Werren & Windsor, 2000). This estimate emerges as the result of several *Wolbachia* screenings, where arthropod, mainly insect species, are tested for infection. In most of the cases, only one individual per species is tested, which we will refer to as one-individual samples. There is one study that gives much higher infection rates of 76% (Jeyaprakash & Hoy, 2000). However, this study used a ‘long PCR’ method that is much more sensitive to trace *Wolbachia* molecules, and therefore environmental contaminants are more likely to be detected. In contrast, most other studies using standard PCR techniques give consistent estimates of infection levels (Table 1).

**Table 1:** Proportion of infected species found among one-individual samples from several *Wolbachia* screenings

	Number of samples	Proportion of infections (%)
Werren & Windsor (2000)	141	20
Werren *et al*. (1995)	139	15
West *et al*. (1998)	53	15
Kikuchi & Fukatsu (2003)	103	31
Nirgianaki *et al*. (2003)	23	0
Tagami & Miura (2004)	20	25
Gotoh *et al*. (2003)	21	0
Total*	547	19
Jeyaprakash & Hoy (2000)	62	73†

* Includes one-individual samples from all 20 studies.

† Differs from 76% because of two species five individuals were tested which are excluded here.

The following problem arises in studies based on a single or a few individuals per species. If an individual is infected, the species is rightly classified as infected. One or a few uninfected individuals, however, result in the classification of this species to be uninfected. This method works when infection frequencies within infected populations are always high. On the other hand, low infection frequencies are reported as well. For instance, Tagami & Miura (2004) found only 3.1% of the Japanese butterfly *Pieris rapae* to harbour *Wolbachia*. The probability of detecting this infected species would obviously have been low if only a single specimen had been tested. Furthermore, infection levels may depend, in part, on the mode of reproductive manipulation induced by *Wolbachia*; for instance, male-killers are expected to occur at lower frequencies (5–50%) within species than those causing cytoplasmic incompatibility (CI) (Hurst & Jiggins, 2000). There is also theoretical (Turelli, 1994; Flor *et al*., 2007) and empirical (Hoffmann *et al*., 1998) evidence that CI-infected individuals can occur at intermediate or low frequencies. Thus, because within-species infection frequencies differ across species, it is assumable that the *c*. 20% infection level found in several studies by testing a few individuals per species is an underestimate.

Here we present a meta-analysis of 20 different studies investigating the frequency of *Wolbachia*, and develop a statistical approach to estimate the overall frequency of *Wolbachia*-infected species. We show that studies where >100 individuals per species were tested tend to be biased towards infected species. Correcting for this bias, we estimate that 66% of species are infected with *Wolbachia*. It should be emphasized that this estimate was not achieved using the approach of Jeyaprakash & Hoy (2000); that study was excluded from the analysis due to its infection estimates being an outlier relative to other samples and to the highly sensitive PCR methods used. Rather, the estimate is derived from studies that routinely give 15–30% infection rates when one individual per species is tested, and extrapolating from these the expected percent of infected species among arthropods.

By applying a beta-binomial model, we can estimate a function describing the distribution of infection frequencies within species, and provide an estimate of the total percentage of infected species. This work aims at investigating to which degree the frequency of *Wolbachia* has been underestimated in previous studies and pointing out sampling methods necessary to obtain estimates of the distribution of *Wolbachia* within and among species.

### Data analysis

We summarized data from 20 different *Wolbachia*-screenings (Werren *et al*., 1995; Breeuwer & Jacobs, 1996; Bouchon *et al*., 1998; West *et al*., 1998; Kondo *et al*., 1999; Plantard *et al*., 1999; Werren & Windsor, 2000; Jiggins *et al*., 2001; Ono *et al*., 2001; Van Borm *et al*., 2001; Shoemaker *et al*., 2002; Vavre *et al*., 2002; Gotoh *et al*., 2003; Kikuchi & Fukatsu, 2003; Nirgianaki *et al*., 2003; Rasgon & Scott, 2003; Rokas *et al*., 2002; Shoemaker *et al*., 2003; Thipaksorn *et al*., 2003; Tagami & Miura, 2004). These 20 studies include data from 9432 individuals of 917 arthropod species.

The data show an increasing frequency of infected species with the number of individuals tested. Part of this trend is likely due to studies with large sample sizes having focused on species already known to be infected to determine infection frequencies within species more precisely (Van Borm *et al*., 2001; Rasgon & Scott, 2003). In contrast, samples comprising predominantly one-individual samples of unknown infection status aimed at determining the overall infection frequency among various arthropod species (Werren *et al*., 1995; Werren & Windsor, 2000). Thus, it does not represent an unbiased sample. We deal with this issue using both the complete data set and supposedly less biased subsets for a statistical analysis to estimate overall species infection frequencies. We then test the different data sets for bias. Another problematic point is that different orders might not be evenly represented by samples due to collection methods. There are some studies that focus on single insect orders; others screen individuals from various species and orders. Obviously, these conditions impair the emerging estimates. Nevertheless, they serve as a first attempt to interpret existing data.

Our goal is to estimate the total proportion of infected species as well as to describe the distribution of infection frequencies within species. Both can be achieved using a beta-binomial model (Böhning, 1999; Carlin & Louis, 2000). The beta-binomial model considers *N* random variables *X*_j_, which are all binomially distributed, but each with different parameters *q*_j_ and *n*_j_, so that *X*_j_∼*Bin*(*q*_j_, *n*_j_). The parameters *q*_*j*_ of the species-specific binomial distributions are assumed to themselves follow a distribution. If this distribution is the beta distribution, the conditions to apply a beta-binomial model are fulfilled.

The beta distribution depends on two parameters α and β, which are to be estimated within the framework of a beta-binomial model [for details, see Böhning (1999); Carlin & Louis (2000)]. To obtain the estimates and thus the distribution of the infection frequency within species, we apply a procedure consisting of the following three steps:

1. Determination of moment estimators $\hat{\text{μ}}$ and $\hat{s}$ by
(1)
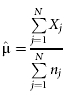

and
(2)
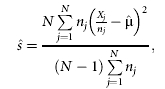

where *X*_*j*_ is the number of infected individuals, *n*_*j*_ is the number of individuals tested of species *j* and *N* is the number of tested species.
2. Determination of α and β by the following equations:
(3)
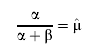

and
(4)
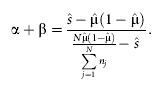
3. Determination of the overall infection rate *x* by integrating the distribution of the infection rates within species, which is a function of both estimated parameters α and β:
(5)
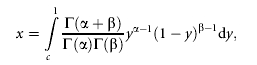

where *c* defines a threshold frequency below which species are considered to be uninfected.

By weighting the infection frequencies within species with the particular sample size [Eqns (1) and (2)], large samples have a strong impact on the estimation procedure. This can be a problem because large samples might be based on prior knowledge and thus not be independent of the parameter being estimated. This is likely the case for the largest sample from *Culex pipiens* (Rasgon & Scott, 2003), of which 1090 individuals were tested (1083 were found to be infected). *Culex pipiens* was known to be infected prior to this survey (Yen & Barr, 1973) and this prior knowledge presumably led to the collection and screening of more than thousand individuals. Among the 13 species with more than 100 individuals tested, 12 harboured *Wolbachia*. This is almost certainly due to the researcher bias of carrying out more extensive sampling of species already known to harbour *Wolbachia* infections (Table 2).

**Table 2:** Proportion of infected species found for different sample sizes

Sample size *n*	Number of samples	Infected species (%)
1	547	19
2	110	21
10	6	33
≥10	115	54
>100	13	92

To test for the potential biases of larger samples, we determined parameter values for three different sample sets, and then tested these for evidence of bias. Specifically, we determined three different distributions *B*_(i)_, *B*_(ii)_ and *B*_(iii)_ based on three different data sets: (i) complete data, (ii) without the *C. pipiens* sample (thus *n*_*j*_<1000) and (iii) only samples with sample size *n*_*j*_<100.

Because some species were known to be infected before sampling, we further evaluated a data set *B*_(iv)_ excluding 12 species that were primarily analysed to determine natural infection frequency or *Wolbachia*-induced modifications of the reproductive system.

## Results and discussion

**Fig. 1:**
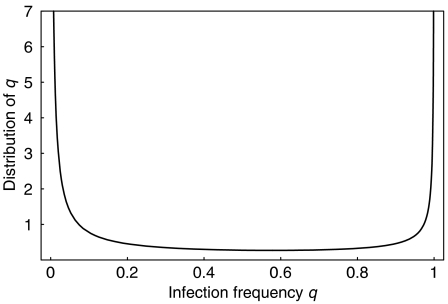
Estimated distribution *B*_(iii)_ of the frequency of *Wolbachia* within species. The underlying data set includes only the samples in which fewer than 100 individuals were tested.

**Fig. 2:**
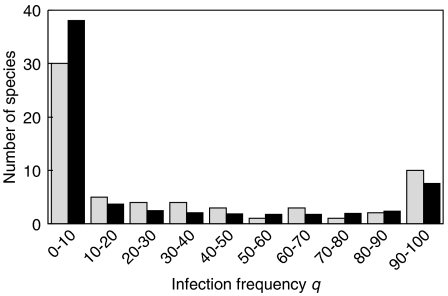
Numbers of species with infection densities in the particular intervals. Gray bars describe the observations made in samples with sample size *n*_j_≥22. The black bars indicate the number of species expected based on *B*_(iii)_. The value of the *χ*^2^- statistic is 8.4 (<14, error probability 5%), thus we can accept this distribution as an underlying density function. Here, also *B*_(i)_ could be accepted, whereas *B*_(ii)_ had to be rejected.

All the resulting functions show a ‘most-or-few’ infection pattern, as very high as well as very low intraspecies infection frequencies are more likely to occur than infection frequencies in between (Figs 1 and 2). Thereby, it should be noted that a beta-distribution can take various forms. Also linear, unimodal or strictly increasing or decreasing functions are possible outcomes within the framework of a beta-binomial model. Further, the weighted average [Eqn. (1)] provides an estimate of the average infection frequency within a species, and an estimate of the overall infection rate is obtained by integrating the beta distributions [Eqn. (5)] from a threshold value *c*, above which species are considered to be infected, up to one (Table 3).

**Table 3:** Estimates of the average infection frequency within species, the parameters α and β and the overall infection rate of *Wolbachia* resulting from different data sets; (i): complete data, (ii) sample size *n*_*j*_<1000, (iii) *n*_*j*_<100

Data set	α	β	Average frequency within species (%)	Infection rate (*c* = 0.001) (%)	Infection rate (*c* = 0.0001) (%)
(i) *B*_(i)_	0.32	0.43	42.8	92.9	96.6
(ii) *B*_(ii)_	0.5	0.9	35.4	97	99
(iii) *B*_(iii)_	0.12	0.36	25.3	65.9	74.2
(iv) *B*_(iv)_	0.18	0.52	26	76.7	84.7

*B*_(iv)_ excludes data from 12 species that were known to be infected. The parameter *c* is the infection frequency above which species are considered infected.

To evaluate which data set is the best candidate to represent *Wolbachia* infection dynamics, we compared certain subsets of the observations (e.g. one-individual samples or large samples only) with expected results, if the estimated distributions were the underlying density functions.

Among the one-individual samples, 104 of 547 species were found to be infected. One-individual samples might represent independent data because species were predominantly randomly chosen, without prior knowledge of the infection status (e.g. Werren *et al*., 1995). Using the *χ*^2^-test, we can check whether our parameter estimates can be accepted as an underlying density function. The weighted average $\hat{\text{μ}}$ of the *n*_j_<100 data set *B*_(iii)_ gives an estimate of the average intraspecies infection rate *q* = 0.253, and the distribution of this model estimates the overall infection rate to be *x* = 0.659 for *c* = 0.001 (or *x* = 0.742 for *c* = 0.0001). Thus, choosing randomly one individual of any species, the probability of obtaining an infected individual is *qx*, where *q* is the average infection frequency within a species. With probability 1−*qx* this individual is uninfected, even though the species might be infected. Based on our estimates, we would expect 547*qx* infected and 547(1−*qx*) uninfected individuals among the one-individual samples. The value of the *χ*^2^-statistic (2.17<3.84, 5% error probability) implies that this is consistent with the observation of 104 infected and 443 uninfected individuals (for *c* = 0.002 this is not consistent; the infection frequency is underestimated). Thus, the estimate for *c* = 0.001 based on *B*_(iii)_ can be interpreted as a lower bound for proportion of infected species estimates.

In contrast, distributions *B*_(i)_ and *B*_(ii)_ are rejected because they overestimate the occurrence of *Wolbachia* (Table 3) in one-individual tested species. This is caused by the high proportion of infected individuals among large samples of species that were probably known to be infected. Including these large samples in the analysis gives estimates of infection frequencies of more than 90% and estimated functions describing intraspecies infection rates that are inconsistent with the one-individual samples. Thus, large samples in fact bias the outcomes towards an overstated number of infected species.

We further compared the observed infection frequencies in species in which at least 22 individuals were tested (by analysing 22 individuals an infection frequency of 10% is detected with a probability of 90%; thus, these samples should represent the distribution of infection frequencies among species) with the expected number of species in certain ranges (Fig. 2) and applied a χ^2^-test. The results confirmed that the beta distribution obtained from the data set excluding large samples (Fig. 1) is a good candidate to represent the underlying distribution of *Wolbachia* infection dynamics (note that this is independent of the parameter *c*).

Data set *B*_(iv)_ yields similar results as *B*_(iii)_, i.e. the resulting function is confirmed by both χ^2^-tests and can thus be considered to be a potential underlying distribution of *Wolbachia* infection frequencies. Here, however, rather low infection frequencies of the influential remaining large samples result in an estimated distribution in which low to intermediate infections occur more prevalently, but these are unlikely to be detected. This yields a higher overall infection frequency estimation (Table 3). For *B*_(iv)_, results from the analysis depend crucially on a few species with large sample sizes within species. Therefore, we conclude that using only *n*_*j*_<100 samples gives the best estimates of the overall percent of infected species.

That the infection rate of *Wolbachia* is likely to be underestimated due to the nondetection of low-frequency infections has been mentioned in several studies (Werren *et al*., 1995; Jiggins *et al*., 2001; Tagami & Miura, 2004). This meta-analysis provides strong support for the proportion of species harbouring *Wolbachia* being in fact significantly higher than 20%. Obviously, these estimates apply primarily to the available data (comprising 904 species after all) possibly not presenting a random choice of species. Further, giving a particular percentage is difficult because the estimator of the overall infection frequency depends on an arbitrary chosen parameter (e.g. *c*). However, we obtained estimates that are consistent with the data from predominantly randomly sampled one-individual samples. Thus, using the above correction, we estimate the total number of infected species to be around 66%. Current estimates of the total number of arthropod species lie between 1 × 10^6^ and 3 × 10^6^, but are more likely in the range of 5 × 10^6^ (Erwin, 1991; Gaston, 1991). The latter estimate implies that a huge number of around 3.3 × 10^6^ species harbour *Wolbachia* infections.

It should be noted that this result does not support the estimate of 76% infected species by Jeyaprakash & Hoy (2000), because our estimation is derived from studies that give predominantly infection rates for one-individual samples of around 20% whereas the Jeyaprakash & Hoy (2000) estimate gives a figure of 76% for predominantly one-individual samples. That study was excluded from this analysis because its one-individual sample estimates of infection are inconsistent with other studies, and their methods are likely more prone to false positives. In contrast, our result is consistent with other one-individual samples (Werren *et al*., 1995; West *et al*., 1998; Werren & Windsor, 2000).

We further conclude that a ‘most-or-few’ infection pattern is likely valid for *Wolbachia*: either very few or most individuals of a species are infected (Figs 1 and 2). Note also that our statistical approach draws attention to the fact that the predicted percent of infected species depends crucially on the minimum cut-off to categorize a species as infected (*c*). If we accept one of 10 000 individuals with an infection as defining an infected species, we will obtain a much different estimate than if we use one of 1000 as a cut-off.

We recognize the limitations of the meta-analysis. Data were collected from different laboratories and often using different *Wolbachia*-specific primers for detection, etc. This is a common issue with meta-analyses. It is encouraging that most larger broad taxon screening studies (e.g. >50 species tested and not limited to a single host taxon) give one-individual infection rates within similar ranges of 15–30%. However, the statistical methods shown here can also be applied as data sets improve and more consistent methods across studies are used. It is important to obtain better estimates of the distribution of infection frequencies within species. Thus, more individuals per species should be assayed for randomly chosen species, because we have shown that data from currently existing large samples bias the outcomes of statistical analyses towards a higher infection frequency of *Wolbachia*. However, caution should be exercised, as there will be a tendency to over-sample common species by this method, as large samples from common species are more easily collected.

With sufficient data, it will also be possible to compare the *Wolbachia* infection patterns among different arthropod taxa, across geographical regions, etc. Furthermore, the statistical method used here can be applied to other infectious agents to estimate species infection frequencies and the frequency distribution of infection levels within species.

## Statement

Re-use of this article is permitted in accordance with the Creative Commons Deed, Attribution 2.5, which does not permit commercial exploitation.
